# Exploring the Factors Affecting Unsafe Antisocial Behaviors of Drivers in Iran: A Qualitative Study

**DOI:** 10.30476/BEAT.2021.87240

**Published:** 2021-01

**Authors:** Farshad Faghisolouk, Sanaz Sohrabizadeh, Hamid Soori, Davoud Khorasani-Zavareh

**Affiliations:** 1 *Department of Health in Disasters and Emergencies, School of Public Health and Safety, Shahid Beheshti University of Medical Sciences, Tehran, Iran*; 2 *Safety Promotion and Injury Prevention Research Center, School of Public Health and Safety, Shahid Beheshti University of Medical Sciences, Tehran, Iran*; 3 *Workplace Health Promotion Research Center (WHPRC), School of Public Health and Safety, Shahid Beheshti University of Medical Sciences, Tehran, Iran*

**Keywords:** Unsafe Antisocial Behaviors, Drivers, Iran, Qualitative study

## Abstract

**Objective:**

To explore the factors affecting unsafe antisocial behaviors of drivers in the context of Iran.

**Methods:**

The interviews were conducted from June to November 2019. A number of 19 participants were selected using purposive sampling method. The data were collected using face to face in-depth semi-structured interviews. Content analysis using Granheim’s approach was applied for data analysis.

**Results:**

Seven categories and 14 sub-categories were extracted from the data**.** The categories included cultural factors, educational and training factors, laws, imitating, substance abuse, awareness and attitude, and psychological problems.

**Conclusion:**

Based on the findings, various cultural, legal, educational, individual and psychological factors affect the unsafe antisocial behavior in driving. Seemingly, such behaviors could increase the chance of death or injury caused by road traffic crashes among the road users and affect social welfare of the citizens and road user’s safety.

## Introduction

Atisocial behavior can be considered as actions that ignore the well-being of others. These behaviors have a wide range from harassment by making loud noises to criminal acts. It seems that economic deprivation, poor parenting of children, Attention deficit hyperactivity disorder (ADHD) and having antisocial families can be effective in formation of these behaviors. People with antisocial behaviors impose a high cost on society. Also, these behaviors create economic costs for such individuals and their victims [1, 2]. Prolonged engagement in antisocial behaviors can increase risk for chronic problems and mortality. Reckless driving leading to injuries is one of the results observed in people with antisocial behavior [3]. The unprecedented rise in the death caused by road traffic crashes (RTCs) has increased the concerns and efforts to identify and control the factors affecting the occurrence of such incidents [4]. Based on the UN report in 2018, road traffic injuries (RTIs) kill around 1.35 million people annually all over the world, which makes it the main cause for death of the people aged 5 to29 [5-7]. The need for driving has led to a rise in inner and inter RTIs with high death rate, making this issue a major public health problem [8]. Regardless of type and intensity, such incidents impose a lot of social and economic problems to the society [9]. Analysis of the death caused by RTCs shows that human agent is a major factor in RTIs [10]. On the other hand, the response on scene was hampered by large numbers of lay bystanders who disrupted rescue and transport procedures and complicated the scene triage process by which patients were evaluated for salvageable injuries [11]. Many of RTCs are the result of reckless driving behavior. Driving behavior is a behavior that the driver selects as a pattern for driving [8]. Thus, intervention in human agent is a crucial factor [12]. Analysis of the statistic of fatal RTIs in Iran reveals that human agent plays a major role in RTIs [13]. Using the vehicle in an antisocial manner leads to a phenomenon known as violence in driving which is a risk behavior in driving [14]. This is an international problem and a lot of countries are struggling with it which comprises a range of intentional behavior [15]. The antisocial behavior in driving creates a potential risk for other drivers [16]. Violent behavior on the road is an instance of antisocial behaviors, and there are hundreds of behaviors of this type, which may be unreported [17]. Such behaviors take various forms, and despite the commonalities among various countries, some of them might be considered specifically antisocial in one country. Therefore, identification of these specific behaviors is significant. These behaviors could cause damages to the society, families and citizens [18]. Although antisocial behaviors and its negative consequences are very important in the driving, but not enough studies have been conducted in this area. Therefore, in order to reduce unsafe antisocial behavior in driving, increasing knowledge about these behaviors is required. For filling this gap, the current study was designed to explore the factors affecting these behaviors in the Iran.

## Materials and Methods

*Design of Study*

Content analysis approach was employed for conducting the study. This approach is useful when existing theory or research literature on a phenomenon is limited [19]. This approach provided the means to explain the concept and the background factors in the occurrence of unsafe antisocial behavior in driving.

*Setting*

This study was conducted in Iran. The context of the study was the traffic police office, administration of roads and urban development, emergency medical service Organization, Department of Transportation and Traffic, Road and Transportation Organization, Taxi driving organization, the Road Research Center, the Safety Promotion and Injury Prevention Research Centers and the health and medical centers. 

*Participant’s Selection*

We used the different participants in the study to achieve data gathering triangulation. The participants included pre-hospital emergency staff, the traffic police officers, and the road traffic injury prevention researchers, health in disasters and emergencies experts, injury prevention and safety promotion experts, traffic engineers, emergency medicine specialists, sociologists, psychologists, psychiatrists, urban planning experts, motor vehicle drivers and the victims of RTCs. The minimum age for the participants was 18, and all of them had a driver’s license and 2 years of driving experience. In this study, 19 participants were selected using purposive sampling method from the relevant institutions and research centers. In order to find the drivers with unsafe behavior, the researchers attended the Traffic Police Mental Health center and extracted the names of the drivers who had negative points in driving and selected the drivers who consented to participate in the study. In order to find the participants with injury experience caused by RTCs, the researchers present to the hospital and health centers, viewed the medical records, and selected them.

*Data Collection*

A total of 19 interviews were conducted. Data were collected using face to face in-depth interviews. The researcher asked each participant “tell me about your experience on the unsafe antisocial behaviors related to driving”. After obtaining the main framework, Data were collected through semi-structured interviews with probing questions such as. What is the role of psychological disorders in the formation of antisocial behaviors in driving? All interviews audio taped and transcribed verbatim. Data saturation was obtained after 18 interviews, and one more interview was conducted to make sure that no new concept will emerge. Each interview lasted between 25 to 55 minutes. The interviews were conducted from June to November 2019. 

*Data Analysis*

Data gathering and data analysis were conducted simultaneously [20]. Granheim’s approach was used for data analysis [21]. At the first stage, the interviews were listened and transcribed. In next stage, the whole material was elicited and compiled as a single text that formed the unit of analysis. In third stage, the text was divided into meaningful units. Fourth, the meaningful units were summarized and labeled with codes. Then, the codes were compared based on the similarity and differences, and the similar codes were placed in each category [22]. The process of data analysis was repeated multiple times and the emerging concepts were checked and reviewed by S.S (Principal Supervisor) and D.KZ(advisor) for consensus [23]. 

*Trustworthiness*

In order to ensure the trustworthiness of this study, four strategies recommended by Schwandt et.al were used [24], which included credibility, confirmability, transferability and dependability. In order to ensure the credibility, triangulation method was conducted using the investigator triangulation as well as peer check which was done by the research team meetings and discussions [25]. Member check was also used with participation of the lead researcher (SS) for the credibility and Confirmability of the data. In addition, the interviews, codes and emerged categories were checked by an expert in both qualitative research and road traffic injury prevention field (D.KZ). Transferability was ensured through detailed description of the subject, participants, data collection and data analysis [26].

*Ethical Approval*

The study was approved by the Ethics Committee of the School of Public Health and Safety in Shahid Beheshti University of Medical Sciences (SBMU) (ethical code: IR.SBMU.PHNS.REC.1398.018). The aims of the study were explained for the participants before conducting the study and they could withdraw from the study at any stage. The informed written consent form was signed by the participants regarding interview recording. They were assured of information confidentiality and anonymity of the participants and their official post or their responsibility.

## Results

The participants were 14 males and 5 females with age ranged from 20 to 60 with a mean age of 36 years (Table 1). The number of initial codes obtained from the interviews was 921. Seven main categories and 14 sub-categories were extracted from the data including: cultural factors (with two sub-categories of cultural lag and cultural weakness), educational and training factors (with two sub-categories of public education weakness and specialized training weakness), laws (with two subcategories of weakness in law enforcement and monitoring and weakness in obeying the laws), imitating (with two sub-categories of family and society), substance abuse (with two subcategories of alcohol abuse and drug abuse), awareness and attitude (with two subcategories of risk perception and false beliefs and misconceptions) and psychological problems (with two subcategories of stress and anxiety, and mental disorders) (Table 2). 

**Table 1:** Demographic characteristics of the participants experiencing unsafe antisocial behaviors in motor vehicle drivers in the context of Iran

**Percentage (%)**	**N=19**	**Participants characteristic**
Gender		
79	15	Male
21	4	Female
Age		
16	3	21-30
21	4	31-40
42	8	41-50
21	4	51-60
Participants role		
63	12	Experts
26	5	Drivers
11	2	RTIs Victims
Educational level		
11	2	Diploma
32	6	Bachelor
37	7	Master of science/Medical Doctor
21	4	PhD/Specialist
Marital Status		
79	4	Single
21	15	Married

**Table 2:** Codes, category and sub-categories of unsafe antisocial behaviors in motor vehicle drivers in the context of Iran

**Selected codes**	**Subcategory**	**Category**
Mismatch of culture and technology	Cultural lag	Cultural factors
Using a modern product in a non-modern society		
Absence of driving culture	Cultural weakness	
Weakness of traffic culture		
Absence of forgiveness culture		
Necessity of cultural development of the country		
Insufficient training at schools	Public education weakness	Educational and training factors
Insufficient training at universities		
Necessity of training by government		
Lack of training classes		
Weakness in driver’s license issuing	Specialized training weakness	
Insufficient training in driving institutions		
Weak law enforcement	Weakness in law enforcement and monitoring	Laws
Severe punishment for crimes		
Absence of monitoring while driving		
Disobeying the driving laws	Weakness in obeying the laws	
Unlawfulness		
Following mother	Family	Imitating
Following father		
Following other drivers	Society	
Child ‘learning from peers		
Following classmates		
Being drunk	Alcohol abuse	Substance abuse
Taking psychotropic drugs	Drug abuse	
Low risk perception	risk perception	Awareness and attitude
Possible risk		
Believing in winning over the others	False beliefs and misconceptions	
Thinking of collisions s as only happening to others		
High anxiety	Stress and anxiety	Psychological problems
Absence of calmness in society		
High stress level in the country		
Boarder character disorder	Mental disorders	
Hyperactivity		
Depression		

*Cultural Lag*

The participants referred to incompatibility between the technology and culture as one of the origins of the formation and development of antisocial behavior in driving. That is, using a modern product and cutting edge technology, are not adjusted in Iran as a developing country. They believed that car manufacturing industry is just a montage industry and we do not have the scientific background in this industry and the culture of using the modern technologies. 

*“All of our behaviors have cultural roots. There is something called cultural lag, which means that the material aspect is emerged before the cultural aspect. Cars are imported but the thought and culture are not imported (Interviewee 4)’’.*

*Cultural Weakness*

Some participants stated that most of the unsafe and antisocial driving behaviors are rooted in cultural issues, introducing cultural weakness as one of the main reasons of the occurrence unsafe antisocial traffic behavior we need to strive for cultural development of traffic culture in our country, because we will only attain a desirable level of driving culture if we develop the culture of the country. 

*“Culturally, we do not take into account the benefit of our community and we only look for personal gain. Our priority is ourselves!” (Interviewee15).*

*Insufficient Public Education*

One of the items referred to by all participants was the public education. All of the participants believed that the required training in citizen rights, socialization skills, different behavior types, and life skills are not provided at the schools and universities.

*“The main reason is the lack of proper education and training. In modern society, the individuals need to receive education and training to take on roles and such training is not given by the families and educational centers and this is the main reason (Interviewee 9)’’.*

*Inadequate Specialized Training*

Likewise, either the drivers’ license applicants have not received proper specialized training, or it has not led to change in behavior of drivers. Driving centers are not updated with the needs of modern society, and they don’t benefit from modern techniques such as simulation methods to increase driving experience of the applicants. 

*“The drivers’ license has turned to just an exam. After that, training is all forgotten (Interviewee 13)”.*

*Insufficient Law Enforcement and Monitoring*

The law has not foreseen all aspects of antisocial behavior in driving to ban or impose severe punishment in case these behaviors occur. Even if the law has predicted these behaviors, there is no monitoring by the responsible organizations, and these laws are not enforced. 

*“We have weaknesses in terms of the laws. Or laws have been passed but we are weak in enforcement. We need stricter laws. Law monitoring is not appropriate (Interviewee 3)”.*

*Inadequate Law Obedience*

While most of the antisocial behaviors have been delineated in the existing laws as crimes and obeying those can prevent from most of the behaviors, the drivers do not obey the traffic laws 

*“In our country and our society there is a law disobedience culture in place, and it is not limited to the traffic laws. (Interviewee13)”.*

*Family*

The participants introduced imitating as one of the main reasons for antisocial traffic behavior. People imitate from other behaviors. One of these cases is children’s that following behaviours of their parents and family members. 

*“When a child, since the early childhood, observes when I drive I roll down the window and insult others or annoy others, well he/she learns. (Interviewee5)”.*

*Society*

Another type of behavior is related to imitating the society in which an individual learns such behavior from his peers or the society members. When individuals observe unsafe antisocial behavior in others, they might attempt it themselves.

*“Well, in a society where there are a lot of such things they learn and imitate. It forms their behavior and gives them negative training (Interviewee 5)”.*

*Alcohol Abuse*

Most of the risky and unsafe antisocial driving behaviors are rooted in alcohol abuse. The participants stated alcohol as one of the risk factors of the unsafe antisocial behavior. So it seems that Alcohol-involved drivers or those with blood alcohol concentrations have more unsafe antisocial driving than other drivers. 

*“Most of our risky behaviors are rooted in alcohol, and we have not focused on it (Interviewee 6).”*

*Drug Abuse*

Many drivers who engage in unsafe driving behaviors have been influenced by some drugs. Most of the drugs taken by an individual can disturb some daily affairs like driving. People need to avoid driving after taking such drugs which are mostly neglected. The use of psychotropic substances affects brain function and leads to impaired driving. 

*‘’ I remember saying that you know how addiction and psychotropic drugs can affect in occurrence of these behaviors. The addicted can do anything behind the steering wheel (Interviewee 17).”*

*Risk Perception*

The drivers were unaware of the extent to which the unsafe and antisocial behavior could increase likelihood of traffic collisions. Most of the motor vehicle drivers including both dangerous drivers and normal drivers were unaware of the consequences of their risky behaviors. 

*“We don’t take our risky behavior seriously. I don’t know why this is the case and people don’t guess how dangerous are their action (Interviewee 6).”*

*False Beliefs and Misconceptions*

Many of the participants considered the beliefs and misconceptions about antisocial driving behavior to be one of the causes of such behaviors. Also, many drivers do not believe that some behaviors are anti-social some behaviors and this is also due to the lack of a correct attitude in this regard. 

*“The person can’t differentiate the behaviors, for example, talking on the phone. Most of the drivers think it’s normal to talk on the phone while driving, but it’s a risky behavior (Interviewee 6). “*

*Stress and Anxiety*

The participants believed that problems such as stress are at a high level in the country, and this factor can affect the drivers’ behaviors directly or indirectly. They also stated that the level of anxiety among motor of vehicles drivers increased. 

*“Psychological problems, stress and anxiety are high in our society, and they are not cure or dealt with (Interviewee 1).”*

*Mental Disorders*

The participating of the study referred to mental disorders as one of the effective factors in the formation of these behaviors. They reported the existence of many mental diseases among people in the community, including drivers.

*“Psychologists and psychiatrists must visit the individual when getting a driver’s license (Interviewee 17).”*

## Discussion

Cultural factors, educational and training factors, laws, imitating and following the other behaviors, substance abuse, awareness and attitude and psychological problems were explored as the important factors affecting unsafe antisocial behaviors among drivers in Iran. 

Based on the findings, one of the anti-social factors was cultural factors and various aspects of the driving culture in the country. The results of a similar study indicate that culture might be stable for the individuals like their personalities. Culture is associated with the abnormal driving behavior [27] that is in line with other previous studies in Iran [28]. Driving behavior is strongly affected by driving culture, and this culture includes the actions, expectations, and the official rules that the driver learns from observing the behavior of others in society. Thus, instead of focusing on the role of the individual in traffic safety, the general safety culture must be developed [29]. Therefore, cultural development and growth can play a major role in decreasing the antisocial unsafe driving behaviors [30]. Other factors affected the emergence of such behaviors are educational and training factors. The results of a study show that professional driving education has an influence on the function of simulated driving, and educated drivers potentially have a safer driving style compared to the uneducated group [31]. Professional driving education has a relationship with developing safety attitudes and decreasing dangerous behaviors. Research indicated the effectiveness of formal education before getting a drivers’ license [32]. Similarly, study conducted in Sweden showed that driver’s license learners who received about 118 hours of supervised experience have 35% less RTCs compared to those who have been educated for 41-47 hours [33]; therefore, it seems that educational programs can reduce the emergence of unsafe antisocial behaviors in driving. However, the gap between education and perception is almost problem in even other related studies [34]. Another important factor is the laws. This factor is included of lack of the stricter laws and disobeying of traffic rules. Various risky driving behaviors such as high speed and using a cell phone have decreased after implementation and enforcement of stricter driving laws [35]. A study in the US showed that, Speed limitation decreases the number of RTCs and as a result, the rates of injuries and deaths; in contrast, lack of speed limitation causes a 15 percent increase in the number of people killed in RTCs [36-38]. A Research indicates that correct use of safety belt decreases the risk of severe injury or death in RTIs by 30 to 50 percent [39]. Hence, setting rules and monitoring their enforcement play an important role in reducing the unsafe antisocial behaviors in driving. The other important factor is imitating and following the other behaviors. Young drivers, who had followed their friends’ driving behaviors, reported riskier driving behaviors because they have perceived that their friends have also had risky driving behaviors [28]. Also, risky driving behavior of young male drivers had a significant relationship with their fathers’ risky driving perception. In addition, young female drivers followed their mothers’ perception of risky driving [40]. Therefore, following parents, and peers, played important role in forming unsafe antisocial behaviors among drivers. Another factor was substance abuse. For example, in a study conducted in 2004 in New York with Title “Long-Term Follow-Up Effects of a School-Based Drug Abuse Prevention Program on Adolescent Risky Driving” students who have trained for drug abuse prevention were less likely to express violence and risky driving behaviors compared to the control group. Drug abuse prevention programs had a direct effect on risky driving behaviors through strengthening the attitudes against drinking alcohol [41]. Another study has acknowledged the abuse of some medicines and drugs as the cause of risky behaviors, malfunctioning and reduction of the driver’s abilities for steering the car [42]. Therefore, it seems that drug abuse also can cause the emergence of unsafe antisocial behaviors among drivers. The other finding was the role of awareness and attitude of the motor vehicle drivers regarding the unsafe antisocial behaviors. In this area, a study can be referred to which showed that the risky behaviors in driving can originate from low awareness in society [28, 43]. Another study showed that the awareness raising plans lead to decrease in RTCs at all levels [44] and the RTCs rate decreases with the increase in knowledge and change in the attitudes of the drivers. There is a significant statistical relationship between safer attitudes and low risk of RTCs [45]. Our findings, which pointed to the lack of awareness as a significant factor in driving unsafe antisocial behaviors, is in line with the findings of another study which suggested that a rise in risk perception is negatively correlated with the risky behavior. In addition, high levels of risk perception for a specific behavior were correlated with lower chance of attempting such behaviors [46]. Therefore, the attitude towards traffic can be a predictor of risky behavior in future [47]. Accordingly, we may be able to reduce the incidence of unsafe antisocial behavior in driving by increasing awareness and change of attitude. Other factor affecting unsafe antisocial behaviors in driving is psychological problems of drivers. Accordingly, a number of studies indicated that mental disorders such as anxiety and depression increase the probability of RTCs [28], and impressively decrease the drivers’ abilities [48, 49]. Thus, psychological issues influence drivers’ behavior. Therefore, it is necessary that some interventions be applied to drivers with psychological problems in order to reduce the negative effects of this factor. 

Various cultural, social, legal, educational, individual, and psychological factors can be influenced the emergence of unsafe antisocial behaviors in motor vehicle drivers. In order to road safety promotion and reducing the social harms caused by unsafe antisocial behaviors in driving, necessary interventions can be conducted based on the factors explored from the study, Such as Safety education in schools, revision of driving license regulations, continuous screening of drivers’ psychological health and enforcement of stricter rules. Developing a conceptual model of the unsafe antisocial behaviors in motor vehicle drivers is highly suggested. Further research is needed to develop a valid and reliable tool for evaluating the different aspects and consequences of the unsafe antisocial behaviors in driving. It is also recommended that future studies investigate the relationship between such behaviors in increasing road traffic collisions.

## Limitations

Legal accessibility to traffic police data to provide specifications of drivers with unsafe driving behaviors is one of the limitations of this research; however, rich data was collected with face to face interviewer with key informants in this field. Another limitation of the research was unwillingness of risky drivers to be interviewed. Principle investigator did try to interview with other informant drivers and collect related data.

## Abbreviations:

RTIs: Road traffic injuries; RTCs: Road traffic crashes.
